# Synthesis and Reactivity of New Aminophenolate Complexes of Nickel

**DOI:** 10.3390/molecules190913603

**Published:** 2014-09-02

**Authors:** Siqi Yu, Huan Wang, Jill E. Sledziewski, Venkata N. Madhira, Cyrus G. Takahashi, Michelle K. Leon, Yulia B. Dudkina, Yulia H. Budnikova, David A. Vicic

**Affiliations:** 1 Department of Chemistry, Lehigh University, 6 E. Packer Ave., Bethlehem, PA 18015, USA; E-Mails: siy212@lehigh.edu (S.Y.); sanni.wangh@gmail.com (H.W.); jillsledz@yahoo.com (J.E.S.); mkl215@lehigh.edu (M.K.L.); 2 Department of Chemistry, University of Hawaii, 2545 McCarthy Mall, Honolulu, HI 96822, USA; E-Mails: sharma.madhira@gmail.com (V.N.M.); cyrus.takahashi@gmail.com (C.G.T.); 3 A.E. Arbuzov Institute of Organic and Physical Chemistry, Kazan Scientific Center of Russian Academy of Sciences, 8 Arbuzov str., Kazan 420088, Russia; E-Mails: dudkina.yu@gmail.com (Y.B.D.); yulia@iopc.ru (Y.H.B.)

**Keywords:** nickel, cross-coupling, coordination chemistry, paramagnetic complexes

## Abstract

New well-defined, paramagnetic nickel complexes have been prepared and characterized by X-ray crystallography. The complexes were found to be active for the cross-coupling of alkyl electrophiles (especially ethyl 2-bromobutyrate) with alkyl Grignard reagents. The ligand architecture in these new complexes could potentially be rendered chiral, opening up future possibilities for performing asymmetric cross-coupling reactions.

## 1. Introduction

The realization of efficient and transition metal catalyzed alkyl-alkyl cross-coupling reactions poses significant challenges. A metal has to activate a bulky and electron rich alkyl electrophile (*versus* sterically less encumbered and less electron rich aryl and vinyl substrates). Transition metal alkyl complexes, which can be intermediates in alkyl-alkyl cross-coupling reactions, are also prone to undesired β-hydrogen eliminations. Finally, it must be taken into account that alkyl halides can react with transition metals by one-electron processes affording alkyl radicals, which then must be tamed to participate in a selective reaction. Despite these inherent obstacles, much progress has been made in recent years in identifying active catalysts for alkyl-alkyl cross-coupling reactions, especially based on nickel [1,2,3,4,5,6,7]. Presently, much focus is dedicated to developing new catalysts for coupling secondary alkyl electrophiles, with the hope that the coupling reactions could be rendered enantioselective [5,6,8,9,10].

**Scheme 1:**
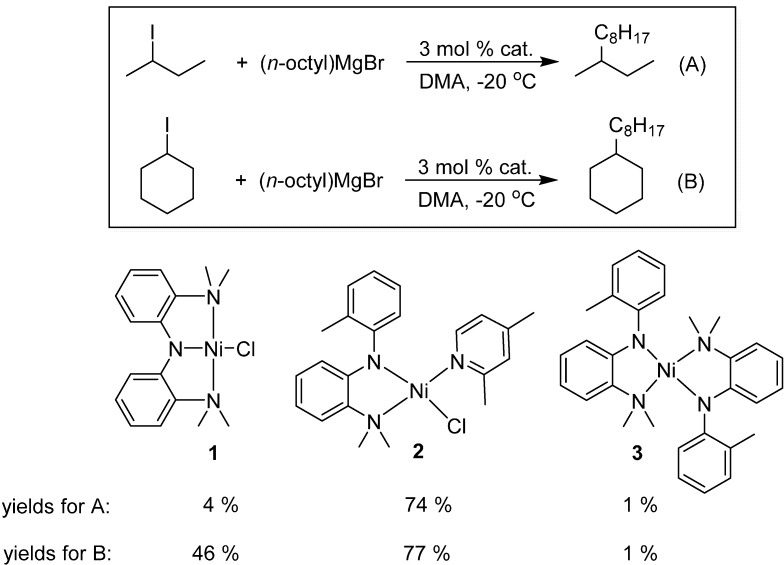
Comparison of amido complexes of nickel for the coupling of secondary alkyl electrophiles.

One interesting development in the coupling of secondary alkyl electrophiles has been the identification of well-defined amido complexes of nickel that mediate Kumada couplings with alkyl Grignard reagents [1,11,12]. It was determined that the nickel pincer complex **1** (Scheme 1) can catalytically couple primary alkyl halides with primary Grignard reagents in excellent yields [12]. However, when this same nickel pincer complex was used to carry out reactions with secondary alkyl electrophiles (Equations (A) and (B) in Scheme 1), yields of cross-coupled product were only 4 and 46%, respectively. This prompted the design of the sterically less encumbered analogue **2** (Scheme 1), which exhibited much higher activity towards secondary alkyl electrophiles [11].

**Figure 1:**
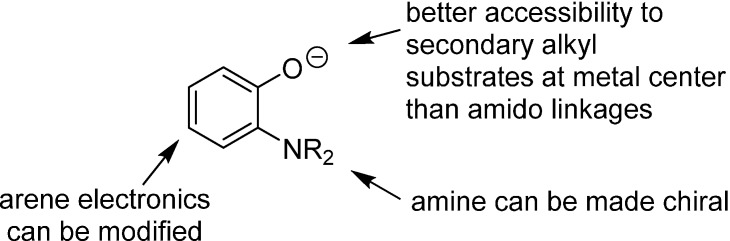
The potential utility of aminophenolate ligands for cross-coupling reactions.

Based on Hu’s results with the second generation catalyst **2**, we imagined that 1,2-aminophenolate ligands (Figure 1) might also be active in alkyl cross-coupling reactions. There are a number of attractive design features in the aminophenolate architecture. The oxygen atom in such a motif is sterically less encumbered than the amido group in **2**, which could afford better accessibility towards secondary alkyl halide substrates. Moreover, the arene electronics of an aminophenolate ligand could potentially be tuned by different substituents. Finally, the aryl amine group could easily be rendered chiral, for instance as a 2,5-dimethylpyrrolidine ligand, for potential asymmetric reactions [13]. With these considerations in mind, we explored the synthesis of new aminophenolate complexes of nickel in order to test their competency in organic cross-coupling reactions.

## 2. Results and Discussion

**Scheme 2:**
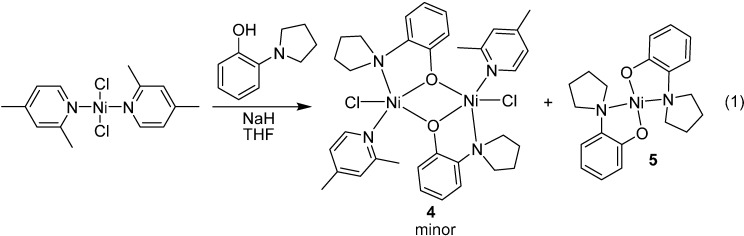
Exploring the coordination chemistry of aminophenol ligands.

Our first efforts to attach an aminophenolate ligand to nickel involved reacting 2-(pyrrolidin-1-yl) phenol with [(2,4-lutidine)_2_NiCl_2_] as described in Scheme 2. The reaction led to complex **4** plus large amounts of the bis-ligated species **5**. Complex **5** was undesired as it has already been shown by Hu that bis-amido complexes like **3** were inactive in organic cross-coupling reactions (Scheme 1).

**Figure 2:**
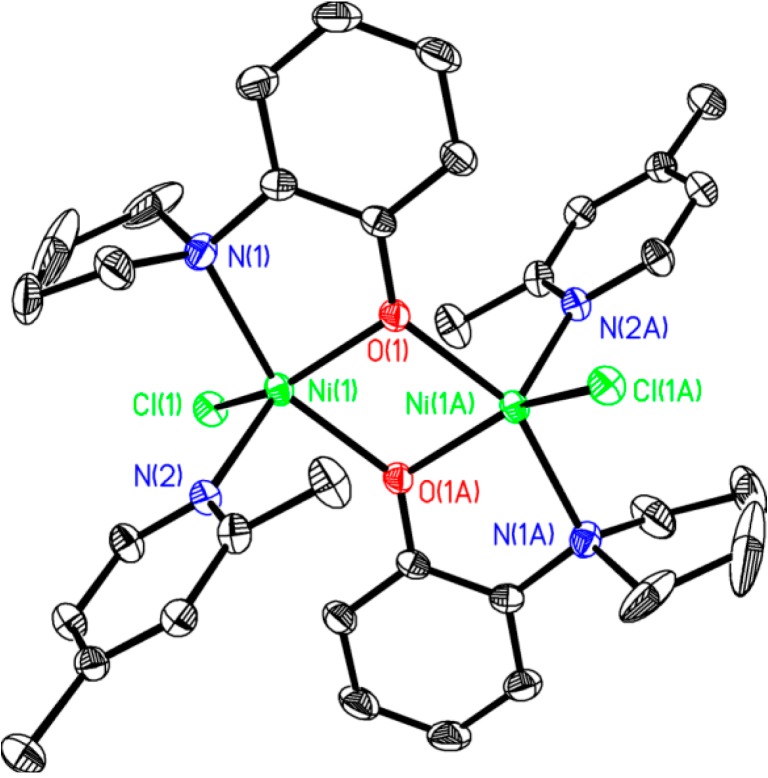
ORTEP diagram of **4**. All hydrogens are removed for clarity. Selected bond lengths (Å): Ni(1)-Ni(1A) 3.2057(6), Ni(1)-Cl(1) 2.3084(11), Ni(1)-O(1) 1.9652(20), Ni(1)-O(1A) 2.065(2), Ni(1)- N(1) 2.211(3), Ni(1)-N(2) 2.053(3). Selected bond angles (°): Ni(1A)-Ni(1)-Cl(1) 121.564(20), Ni(1A)-Ni(1)-O(1) 38.41(6), Ni(1A)-Ni(1)-O(1A) 36.24(5), Ni(1A)-Ni(1)-N(1) 119.26(7), Ni(1A)-Ni(1)-N(2) 110.21(8), Cl(1)-Ni(1)-O(1) 136.39(7), Cl(1)-Ni(1)-O(1A) 97.09(6), Cl(1)-Ni(1)-N(1) 99.93(8), Cl(1)-Ni(1)-N(2) 102.09(7), O(1)-Ni(1)-O(1A) 74.648(19), O(1)-Ni(1)-N(1) 80.86(9), O(1)-Ni(1)-N(2) 120.77(10), O(1A)-Ni(1)-N(1) 155.49(9), O(1A)-Ni(1)-N(2) 92.84(9), N(1)-Ni(1)-N(2) 100.64(10), Ni(1)-O(1)-Ni(1A) 105.35(8).

Poor solubility of [(2,4-lutidine)_2_NiCl_2_] most likely plays a role in the formation of **5** and the attachment of two aminophenolate ligands to the nickel center. While dimer **4** was only formed in minor amounts, red orange crystals of **4** could be separated from the yellow/beige **5** under a microscope in order to investigate its structure by X-ray crystallography. The solid-state structure of **4** is provided in Figure 2. The X-ray data revealed that the environment around the oxygen atom of the ligand is not bulky enough to prevent dimerization, even with coordinated lutidines remaining on both of the nickel centers. Because we could not efficiently purify complexes **4** and **5** from the reaction mixture in bulk, the method for attaching an aminophenolate ligand to nickel as described in Scheme 2 was ultimately abandoned.

**Scheme 3:**
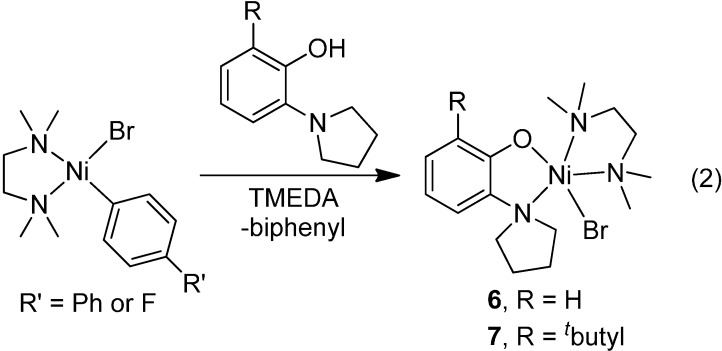
Preparation of monomeric aminophenolate complexes of nickel.

**Figure 3:**
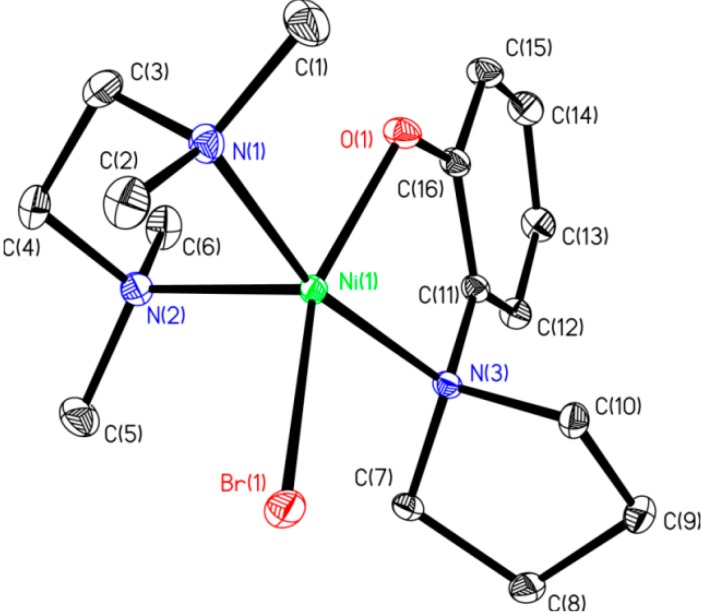
ORTEP diagram of **6**. All hydrogens are removed for clarity. Selected bond lengths (Å): Ni(1)-O(1) 1.9692(6); Ni(1)-N(1) 2.1465(7); Ni(1)-N(2) 2.1088(8); Ni(1)-N(3) 2.1485(7); Ni(1)-Br(1) 2.48086(15). Selected bond angles (°): O(1)-Ni(1)-N(2) 101.01(3); O(1)-Ni(1)-N(1) 87.10(3); O(1)-Ni(1)-Br(1) 157.01(2); N(1)-Ni(1)-N(3) 165.56(3); O(1)-Ni(1)-N(3) 80.83(3).

With the knowledge that another coordinating ligand may be necessary to prevent dimerization of the nickel precatalysts, we explored the use of chelating amines to replace lutidine as the ancillary ligand on the nickel complexes. We found that the most reliable method to generate a monomeric complex bearing aminophenolates was by adding the aminophenol ligand to [(TMEDA)NiBr(Ar)] (Scheme 3). In this way, the nickel product that is obtained is a five-coordinate monomer with good solubility in organic solvents. Complex **6** exhibits a magnetic moment with µ_eff_ (Evan’s method) = 3.02 µB, consistent with a paramagnetic *S* = 1 ground state. The electrochemistry of **6** in DMF is complicated, having multiple irreversible oxidations beginning at −0.05 V (versus Ag/AgNO_3_) and irreversible reductions beginning at −2.07 V. The latter value represents a redox potential more negative than that seen for **1** [14]. Complex **6** crystallizes from THF/pentane, and the ORTEP diagram of **6** is provided in Figure 3.

**Scheme 4:**
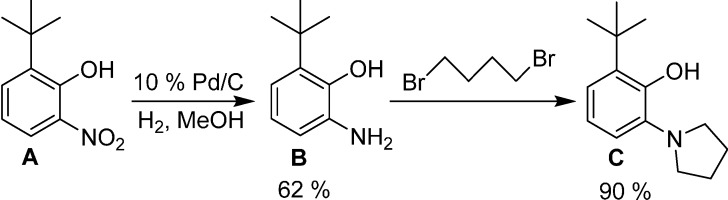
Synthesis of *tert*-butyl-substituted aminophenol ligand **C**.

**Figure 4:**
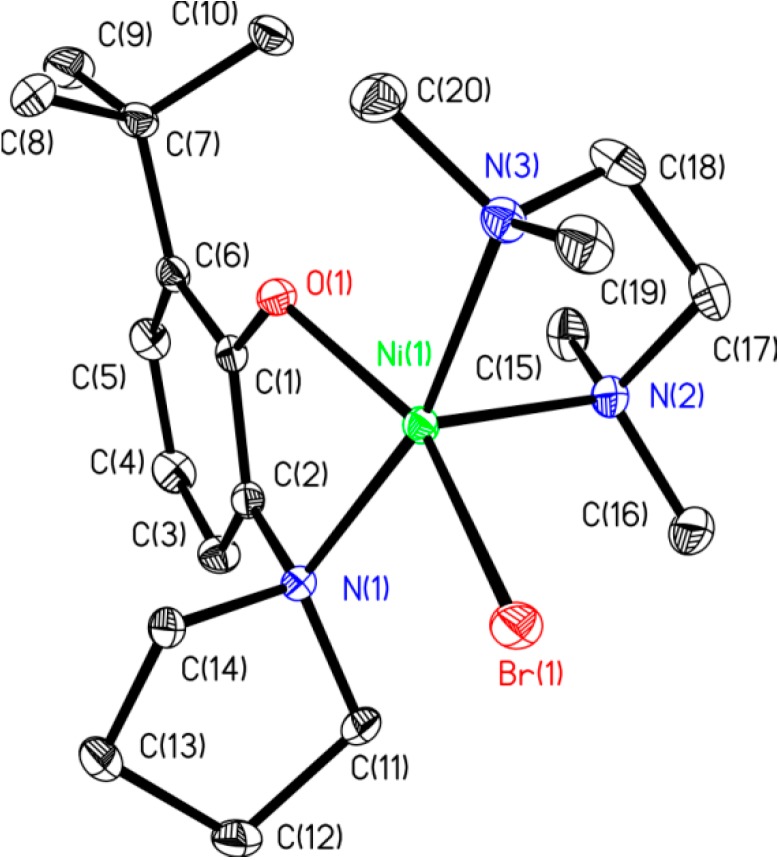
ORTEP diagram of **7**. All hydrogens are removed for clarity. The one non-disordered molecule of the asymmetric unit is shown. Selected bond lengths (Å): Ni(1)-O(1) 1.9584(17); Ni(1)-N(1) 2.138(2); Br(1)-Ni(1) 2.4392(4); Ni(1)-N(2) 2.091(2); Ni(1)-N(3) 2.198(2). Selected bond angles (°): O(1)-Ni(1)-N(2) 103.73(8); O(1)-Ni(1)-N(1) 79.73(7); O(1)-Ni(1)-Br(1) 156.03(5); N(1)-Ni(1)-N(3) 166.55(8); O(1)-Ni(1)-N(1) 79.73(7).

Complex **7**, where the arene ring of the aminophenolate ligand is derivatized with a *tert*-butyl group, was also prepared. The protocol for the *tert*-butyl-substituted ligand synthesis is outlined in Scheme 4. Although the *tert*-butyl-substituted aminophenol precursor **B** has been mentioned in the literature [15,16,17], there are no reports of its spectral characterization. NMR data for **B** is provided in this manuscript. Consistent with its classification as an anti-oxidant [15], **B** is air sensitive, and solutions of **B** quickly turn to dark red upon exposure to air. We found that pure, colorless crystals of **B** can be obtained through sublimation under high vacuum. The target ligand **C** was prepared in excellent yields by reacting **B** with 1,4-dibromobutane under refluxing conditions in ethanol (Scheme 4). Ligand **C** can be complexed to nickel to afford **7** as described in Scheme 3, and an X-ray crystal structure (Figure 4) confirmed the nature of the bonding in the monomeric species.

With complexes **6** and **7** in hand, the aminophenolate complexes of nickel were screened for activity in Kumada-like coupling reactions (Table 1) using *n*-octylmagnesium chloride as the Grignard reagent in order to draw comparisons with the Hu system. Hu’s first generation catalyst **1** was reported to couple the primary alkyl iodide [Ph-C_2_H_4_-I] with *n*-octylMgCl to afford [Ph-C_2_H_4_-oct] in 85% yield, while his second generation catalysts afforded a maximum yield of 41% [11].

**Table 1:** Efficiency of alkyl-alkyl coupling reactions with aminophenolate complexes of nickel *^a^*. 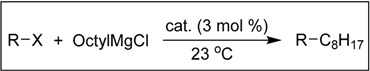

Entry	Substrates	Product	Solvent	Yield (%) using 6	Yield (%) using 7
1	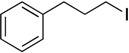	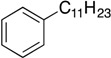	THF	75 *^b^* (78 *^c^*)	72 ^c^
2	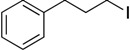	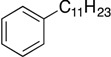	Dioxane	68 *^b^*	57 ^c^
3	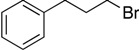	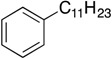	THF	34 *^b^*	25 ^c^
4	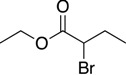	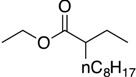	THF	78 *^c^*	89 ^c^
5	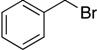	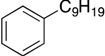	THF	40 *^c^*	88 ^c^
6	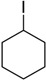	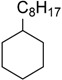	THF	15 *^c^*	10 ^c^

*^a^* Reaction conditions: organic halides (0.2 mmol), octylMgCl (0.24 mmol) and the nickel(II) catalyst (3 mol %), THF (3 mL); *^b^* isolated yield; *^c^* GC yield.

Our catalysts **6** and **7** were able to couple similar primary alkyl iodides in 78% and 72%, respectively (Table 1, entry 1). THF proved to be a slightly better solvent for the reaction that dioxane (Table 1, entry 2). Primary alkyl bromides were not good substrates for our new catalysts (Table 1, entry 3). Gratifyingly, catalysts **6** and **7** gave excellent yields for the coupling of ethyl 2-bromobutyrate to cross-coupled product in 78% and 89%, respectively (Table 1, entry 4), suggesting that aminophenols are promising ligand scaffolds for the cross-coupling of alpha-bromoketones. Complex **7** was also able to efficiently cross-couple benzyl bromide (Table 1, entry 5) although moderate yields were obtained with catalyst **6**. Although Hu’s second generation catalyst **2** was able to cross-couple iodocyclohexane in 77% yield [11], our catalysts performed poorly with this substrate, only affording product in less than 15% yield (Table 1, entry 6).

## 3. Experimental Section

### 3.1. General Information

All manipulations were performed using standard Schlenk and high vacuum techniques or in a nitrogen filled glovebox. Solvents were purified by passing through activated alumina and/or copper in a solvent purification system supplied by Pure Process Technology. Solution ^1^H-NMR spectra were recorded at ambient temperature on a Bruker DRX 500 MHz spectrometer and referenced to residual proton solvent signals. ^13^C-NMR spectra were recorded on a Bruker NMR spectrometer operating at 125 MHz and referenced to solvent signals. ^19^F spectra were recorded on the Bruker NMR spectrometer operating at 470 MHz and referenced to trifluorotoluene set at δ −63.7. A Bruker D8 Quest diffractometer was used for X-ray crystal structure determinations. Elemental Analyses were performed at Midwest Microlab, LLC. Cyclic voltammograms were recorded with a BASi Epsilon potentiostat (USA) at room temperature in DMF solution with [Bu_4_N][BF_4_] (0.1 M) as the supporting electrolyte. The substrate concentration was 10^−2^ M. A glassy-carbon (3.0 mm dia.) electrode was used as the working electrode. The auxiliary electrode was a platinum rod. The scan rate was 100 mV s^−1^. Crystallographic data (excluding structure factors) for compounds **4**, **6**, and **7** have been deposited with the Cambridge Crystallographic Data Centre as supplementary publication numbers CCDC 1003595, 1003594, and 1003589, respectively. Copies of the data can be obtained free of charge on application to CCDC, 12 Union Road, Cambridge CB2 1EZ, UK [fax: +44 1223 336 033; E-Mail: deposit@ccdc.cam.ac.uk].

### 3.2. Preparation of [(TMEDA)Ni(Br)(C_6_H_4_F)]

TMEDA (166 mg, 1.43 mmol) and 1-bromo-4-fluorobenzene (250.25 mg, 1.43 mmol) were dissolved in toluene (8 mL). Ni(COD)_2_ (400 mg, 1.43 mmol) was then added and the resulting mixture was stirred at room temperature for 3 h. A red orange solid precipitated and was washed with pentane. The product was then dried under high vacuum line to yield an orange power (yield 75%). Anal. Calcd. (found) for C_12_H_20_BrFN_2_Ni: C, 41.19 (40.90); H, 5.76 (5.82). ^1^H-NMR (500 MHz, THF, δ): 7.59 (d, *J* = 8.0 Hz, 2H), 7.47–7.39 (m, 2H), 6.60–6.39 (m, 2H), 2.77–1.94 (br, 16H). ^19^F-NMR (500 MHz, THF, δ): −127.37.

### 3.3. Preparation of [(TMEDA)Ni(Br)(2-(1-pyrrolidinyl)phenol)] (**6**)

2-(1-Pyrrolidinyl)phenol (0.142 g, 0.87 mmol) and [(TMEDA)NiBr(C_6_H_4_Ph)] [18] (0.308 g, 0.75 mmol) were dissolved in THF (20 mL) and stirred at room temperature for 16 h. The mixture was then filtered to give a clear red orange filtrate. The solvent was then removed on a high vacuum line giving a green solid. The solid was washed using copious amounts of pentane and dried under vacuum. Yield: (0.235 g, 75%). Anal. Calcd (found) for C_16_H_28_BrN_3_NiO: C, 46.08 (46.21); H, 6.77 (6.66). Product is paramagnetic. µ_eff_ (Evan’s method) = 3.02 µB.

### 3.4. Preparation of 2-Amino-6-(tert-butyl)phenol

2-(*tert*-Butyl)-6-nitrophenol [19] (2 g, 10.2 mmol) was dissolved in anhydrous methanol (40 mL) in a Parr hydrogenator glass vessel. To this, 10% Pd/C (0.10 g, 0.1 equiv.) was slowly added. The hydrogen gas pressure was adjusted to 40 psi on the Parr hydrogenator. This reaction mixture was allowed to shake for 16 h, and then the volatiles were removed under vacuum. The dried mixture was then taken into glovebox, dissolved in THF, and filtered through Celite. The volatiles were removed under vacuum to yield white powder, which could be sublimed under reduced pressure. Yield: 1.04 g, 62%. ^1^H-NMR (500 MHz, chloroform-*d*) δ 6.91 (d, *J* = 7.8 Hz, 1H), 6.81 (d, *J* = 7.6 Hz, 1H), 6.74 (t, *J* = 7.8 Hz, 1H), 4.22 (s, 2H), 1.42 (s, 10H). ^13^C-NMR (500 MHz, toluene-*d_8_*) δ C, 34.82, CH, 146.7, 138.1, 134.1, 120.4, 118.4; CH_3_, 29.5.

### 3.5. Preparation of 2-(Tert-butyl)-6-(pyrrolidin-1-yl)phenol

2-Amino-6-(*tert-*butyl)phenol (0.165 g, 1.0 mmol) was dissolved in toluene (10 mL), followed by addition of 1,4-dibromobutane (0.215 g, 1.0 mmol) and *N*,*N*-diisopropylethylamine (1.2 g, 3 equiv.). The solution was then refluxed for 36 h under the protection of nitrogen gas. Then the reaction mixture was cooled to room temperature, washed with water and brine solution, and dried over sodium sulfate. The volatiles were removed under vacuum to yield the desired compound. Yield: 219 mg, 90%. ^1^H-NMR (500 MHz, chloroform-*d*) δ 7.09 (dt, *J* = 7.9, 1.4 Hz, 3H), 6.81 (td, *J* = 7.9, 1.3 Hz, 1H), 3.01 (s, 4H), 1.99 (s, 4H), 1.44 (s, 10H). ^13^C-NMR (500 MHz, chloroform-*d*) δ C, 34.74, CH, 151.58, 137.26, 134.91, 123.40, 119.04, 118.73; CH_2_, 50.02, 24.58; CH_3_, 29.53.

### 3.6. Preparation of Complex **7**

2-(*tert*-Butyl)-6-(pyrrolidin-1-yl)phenol (109 mg, 0.5 mmol) was dissolved in THF (4 mL). (TMEDA)NiBr(4-fluorobenzene) (175 mg, 0.5 mmol) was dispersed in THF (6 mL). The solution of 2-(*tert*-butyl)-6-(pyrrolidin-1-yl)phenol was then added slowly into the solution of (TMEDA)NiBr(4-fluorobenzene), and the resulting mixture was allowed to stir for 5 h. All volatiles were then removed on a high vacuum line. The residue was dissolved in a minimal amount of THF and solids were precipitated with pentane. The solids were filtered with a fine frit and discarded. The yellow filtrate was pumped dry to yield greenish yellow solid. Yield 40% of paramagnetic material. µ_eff_ (Evan’s method) = 2.71 µB. Anal. Calcd (found) for C_20_H_36_BrN_3_NiO: C, 50.77 (48.91); H, 7.67 (7.99).

### 3.7. Typical Procedure for the Kumada Cross-Coupling Reactions in Table 1

A vial was charged with alkyl halide substrate (0.2 mmol) and nickel catalyst (3 mol %), and the internal standard undecane (0.2 mmol) in THF (3 mL). OctylMgCl (0.24 mmol, 2 M in THF) was then added dropwise at room temperature. The resulting solution was stirred at room temperature for 2 h. The reaction mixture was then filtered through a plug of silica gel and analyzed by gas chromatography. The response factor of ethyl undecanoate calibrated to the internal standard was used to calculate product yields.

## 4. Conclusions

New well-defined, paramagnetic nickel complexes have been prepared and characterized by X-ray crystallography. The complexes were found to be active for the cross-coupling of alkyl electrophiles (especially ethyl 2-bromobutyrate) with alkyl Grignard reagents. No β-hydride elimination products were observed in the cross-coupling reactions. The only other identifiable side-product in all reactions was reduced alkane, suggestive of single electron radical pathways being operative under the reaction conditions. The ligand architecture in these new complexes could potentially be rendered chiral, opening up future possibilities for performing asymmetric cross-coupling reactions.
